# Investigating memory episodes in location probability learning: Can altering response features reset spatial bias?

**DOI:** 10.3758/s13414-025-03106-6

**Published:** 2025-06-11

**Authors:** Xinger Yu, Geoffrey F. Woodman

**Affiliations:** Department of Psychology, Vanderbilt University, 301 Wilson Hall, 111 21St Ave South, Nashville, TN 37240 USA

**Keywords:** Statistical learning, Probability cueing, Episodic retrieval, Motor response

## Abstract

When individuals are repeatedly exposed to a specific task, they store each occurrence as a distinct memory episode, which includes stimuli, responses, and outcomes. The accumulation of these episodes enables more efficient retrieval over time, particularly under consistent conditions, leading to quicker and more automatic responses. This mechanism likely underlies the statistical learning effect observed in tasks such as the probability cueing paradigm, where frequent target detection at a predictable location enhances performance through rapid, less conscious retrieval of relevant episodes. In this study, we explored the role of episodic retrieval in implicit location probability learning, focusing specifically on how changes in response features might impact the retrieval of memory episodes and, consequently, the learned attentional bias. Participants performed a visual search task, searching for a T among Ls, while unaware that the target was more often in one screen region. An attentional bias towards this region developed during training. In the testing phase, we examined whether changes in motor responses could negate the learned attentional bias, as such changes might prevent the retrieval of relevant memory episodes. Contrary to our initial hypothesis, two experiments showed that changing response features did not affect the attentional bias. This study expands our understanding of human statistical learning by examining the previously neglected yet increasingly recognized role of episodic retrieval in shaping implicit learning processes, thereby opening new avenues for understanding how our visual systems adapt to and learn from the environment.

**Supplementary Information:**

The online version contains supplementary material available at 10.3758/s13414-025-03106-6.

## Introduction

Humans live in a complex visual environment, containing a massive amount of information that exceeds what one can perceive and respond to at any given moment. The ability to extract regularities from the environment to support automatic behavior is critical and is often referred to as *statistical learning*. Research over the past few decades has consistently confirmed this learning ability regarding environmental regularities across various cognitive domains and modalities (Frost et al., 2015, 2019). For example, after learning, attention can be optimally biased towards locations that are most likely to contain targets during visual search, leading to faster target detection at these locations (Geng & Behrmann, 2001, 2005; Golan & Lamy, 2023; C. Huang et al., 2022; Jiang et al., 2013; Zhang & Carlisle, 2023). Importantly, statistical learning is assumed to be an implicit process that requires neither the intention to learn nor the explicit awareness (Duncan & Theeuwes, 2020; Turk-Browne, 2012; Turk-Browne et al., 2005).

A classic paradigm for studying how statistical learning influences visual attention is known as the *location probability cueing paradigm* (Geng & Behrmann, 2001, 2005). Jiang et al., (2013, 2014), for example, instructed participants to search for a letter T among Ls. Unbeknownst to the participants, the target T was placed in one rich quadrant for 50% of the trials and distributed equally across the remaining three sparse quadrants for 16.7% of the trials each. Participants’ reaction times (RTs) were faster when the target appeared in the rich quadrant compared to the sparse quadrants. Moreover, their initial saccades were twice as likely to be directed towards the rich quadrant, indicating that probability cueing is grounded in an early attentional effect. Importantly, these effects manifest regardless of the participants’ awareness of the spatial regularities (Gao & Theeuwes, 2022; Turk-Browne et al., 2005), indicating that probability cueing represents a form of selection history that does not depend on top-down attentional control.

Besides its implicit nature, another defining characteristic of statistical learning is its enduring effect. Jiang et al., (2013, 2014) demonstrated that the spatial attentional bias induced by probability cueing persisted for hundreds of trials after the targets were distributed evenly and could still be observed after a week’s delay (see also Golan & Lamy, 2023). This durability distinguishes statistical learning from low-level intertrial repetition priming (Kristjánsson & Driver, 2008; Maljkovic & Nakayama, 1994), which typically exhibits a short-term nature in timing. Furthermore, the learning of attentional bias does not depend on the availability of spatial working memory resources (Gao & Theeuwes, 2020b; Won & Jiang, 2015) and is not affected by aging (Jiang et al., 2016). Taken together, these findings suggest that our visual system automatically encodes and retrieves information from the environment that is relevant to the task at hand.

The canonical account of statistical learning is framed as a flexible weighting within a spatial priority map (Ferrante et al., 2018). According to this account, whenever attention is directed towards a target, an implicit memory association between the target and its location is formed (Chun & Nakayama, 2000). This association subsequently influences the deployment of attention in future trials by increasing the priority weight of that location (Theeuwes et al., 2022). However, our research explores an alternative hypothesis for understanding statistical learning, known as the *episodic-retrieval account* (Hillstrom, 2000; L. Huang et al., 2004; Lamy et al., 2010). Essentially, various elements of a search trial, such as the presentation of the target at a specific location and the behavioral response that was performed, are integrated into a comprehensive memory episode (Logan, 1988), or an event file (Hommel, 1998; Kahneman et al., 1992). When searching for the same target again, the brain retrieves these previous instances of the task (Frings & Rothermund, 2011; Frings et al., 2007), using it as a shortcut (Henson et al., 2014) to quickly locate the target and respond. Thus, an important prediction of the episodic-retrieval account is that the behavioral response is part of the bound representations that are stored in memory, otherwise learning would not support the more efficient response selection that is observed as people become automatic at a task (Logan, 2002).

The episodic-retrieval account has primarily been invoked to explain intertrial repetition priming in visual search (Hilchey et al., 2018; L. Huang et al., 2004; Lamy et al., 2010; Logan, 1990; Neill, 1997). Recently, Theeuwes et al. (2024) extended this concept by demonstrating the existence of a binding and retrieval process when participants learned to associate specific motor responses with target locations. For instance, when a target appears in a specific location, the frequently performed action in response (such as pressing a button with the right index finger) becomes associated with that location, leading to faster responses for that specific action-location pairing. This finding suggests that the features of the stimulus (e.g., location) and the response are integrated into a single memory episode (Moeller et al., 2016). Consequently, attending to a location can automatically retrieve its corresponding response information, improving the search efficiency.

Our study aimed to delve deeper into the episodic-retrieval model by examining how changes in response features may impact the retrieval of memory episodes and, consequently, the learned attentional bias. A recent investigation by Zhang and Carlisle (2023) demonstrated that changing the target itself could disrupt the spatial bias developed during training, suggesting that modifications to target-defining features within the memory episode can alter statistical learning. However, the effects of altering task-irrelevant response features, such as the specific motor action taken, remain underexplored. We hypothesize that if statistical learning hinges on the episodic retrieval of a previous solution in memory, then simply changing the motor response could disrupt this automatic retrieval mechanism, leading to a reduction in attentional bias.

**Fig. 1:**
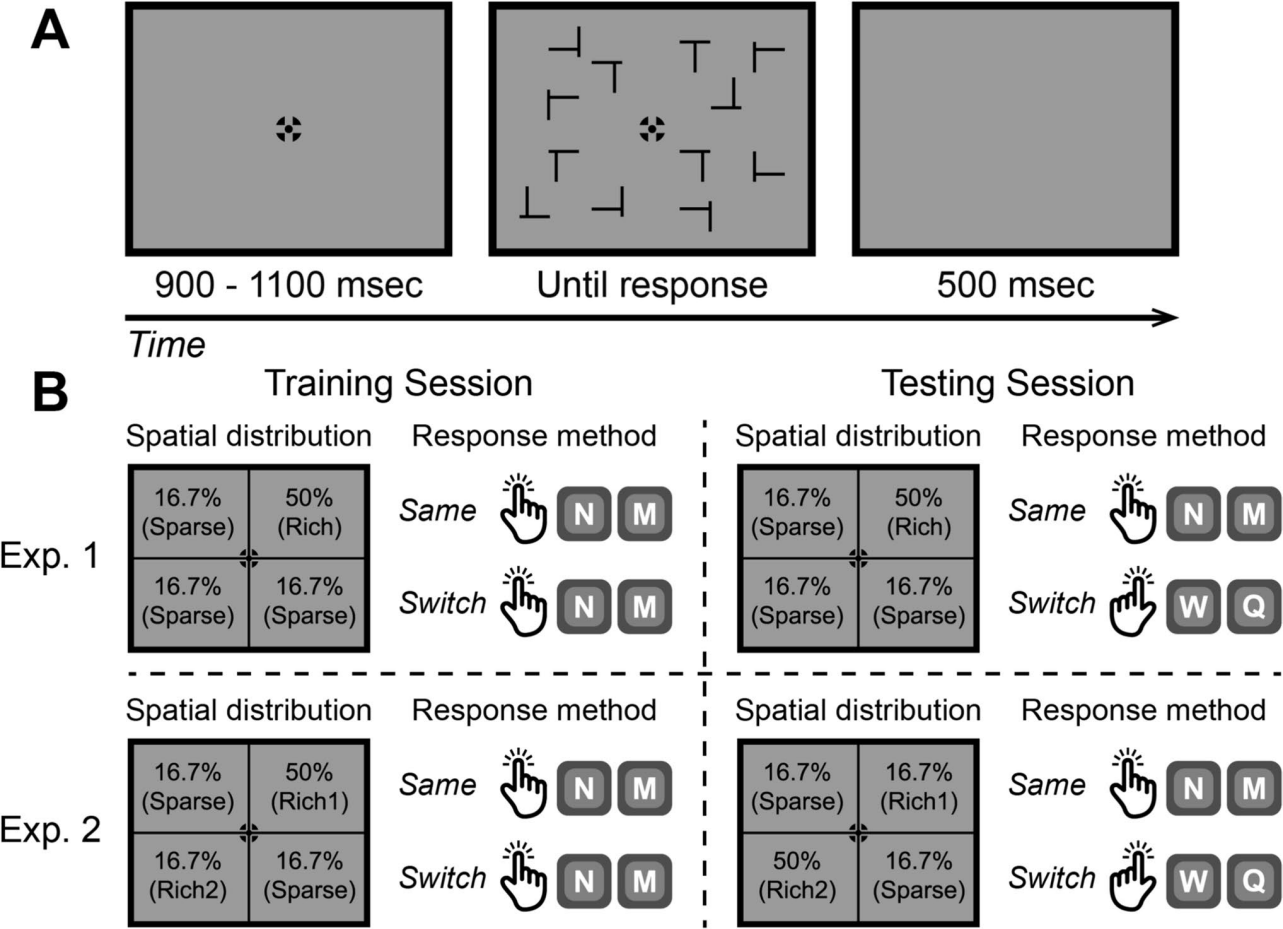
(**A**) This panel illustrates a sample visual search trial in which participants searched for a T among Ls, reporting the orientation of the T. Each quadrant contained three items. (**B**) This panel outlines the spatial distribution of targets and the response method employed throughout the training session (left) and the testing session (right). During the training phase, the target was located in a specified quadrant (illustrated as the upper right) on 50% of the trials and distributed evenly across the other three quadrants on 16.7% of trials each. Participants were instructed to press key N with their right index finger for a T oriented at 0° and key M with their right middle finger for a T oriented at 180°. The key manipulation took place during the testing phase. In Experiment 1, the uneven spatial distribution of targets was maintained. Participants within the *same* group continued using their original response method. Conversely, participants in the *switch* group were required to alter their response configuration, now using the left index finger for key W (for a T at 0°) and the left middle finger for key Q (for a T at 180°). In Experiment 2, the spatial distribution of targets was adjusted so that the quadrant rich in targets during training became sparse, with the previously sparse quadrant (diagonally opposite) becoming target-rich. Similar to Experiment 1, participants in the *switch* group were instructed to change their response hands and keys for the testing phase, while those in the *same* group retained their initial method

To this end, we adapted the probability cueing paradigm from Jiang et al. (2013) such that the target appeared more frequently in one region than in others (Fig. 1). In our experiments, participants were tasked with identifying a T among 11 Ls. During the training session, the target T was placed in the rich quadrant in 50% of the trials and was distributed equally across the other quadrants for the remaining trials. Participants were instructed to use a specific response configuration to report; for example, they were to use the right index finger to press key N for a T oriented at 0° and the right middle finger to press key M for a T oriented at 180°. The critical manipulation occurred during the testing phase. In Experiment 1, the uneven spatial distribution of targets was maintained. Participants in the *same* group continued with their original response requirement, while those in the *switch* group changed their response configuration. The *switch* group now used the left index finger to press key W for a T oriented at 0° and the left middle finger to press key Q for a T oriented at 180°. If statistical learning results from the automatic retrieval of past solutions from memory, we would expect to see a rapid decline in the search advantage for the rich quadrant as soon as the *switch* group changed their response keys. In Experiment 2, the uneven spatial distribution of targets was altered; the quadrant that was rich during the training session became sparse, and the quadrant that was previously sparse, diagonally opposite, became rich. We hypothesized that if changing response configuration facilitated the formation of a new association between the now-prioritized rich quadrant and its response characteristics, then the *switch* group would be more effective in reallocating spatial attention towards this newly rich quadrant.

## Experiment 1

In Experiment 1, participants searched for a T among 11 Ls. During the training phase, the target T was more likely to appear in one quadrant 50% of the time, making it three times more likely than in the other quadrants. Participants were instructed to press specific keys based on the T’s orientation. This response requirement was maintained in the test phase for the *same* group, whereas the *switch* group altered their response configuration. The aim of the study was to determine whether changing response features would affect the speed at which participants found the target, thereby suggesting that statistical learning stems from the automatic retrieval of solutions from memory.

### Material and methods

#### Participants

Forty-eight undergraduates from Vanderbilt University participated in this study for course credit (38 self-reported females, 10 males; age range: 18–24 years). They were randomly assigned to either the *same* or *switch* groups. We chose a sample size of 24 for each group to match that of Experiment 5 in Zhang and Carlisle (2023), where a reset in learned attentional bias was observed with changing search targets. All procedures received approval from the Vanderbilt University Internal Review Board. Furthermore, all participants provided written consent after being informed and reported having either normal or corrected-to-normal vision.

#### Apparatus

Stimuli were presented on an ASUS VG248 monitor, with a spatial resolution of 1,920 × 1,080 pixels and a refresh rate of 60 Hz. The presentations were managed using MATLAB software (The MathWorks, Natick, MA, USA) with the Psychophysics Toolbox (Version 3.0.18; Brainard, 1997; Pelli, 1997). Participants viewed the monitor from a distance of 93 cm in a dimly lit, sound-attenuated room. Participants’ head positions were stabilized using a chin rest. Eye movements were monitored using a video-based eye-tracking system (Eyelink 1000 plus; SR Research, Kanata, Ontario, Canada), which recorded from the dominant eye at a sampling rate of 1,000 Hz.

#### Stimuli

Each search stimulus consisted of a horizontal and a vertical line, each measuring 1° × 0.1°. The target, T, featured a vertical line attached to the center of the horizontal line and could rotate to either 0° or 180°. The distractor, L, had its vertical line connected at either the 1/4 or 3/4 point of the horizontal line and could rotate to 0°, 90°, 180°, or 270°. The search display contained 12 items: one T and 11 Ls (Fig. 1A). These were randomly placed within an invisible 10 × 10 matrix of 15° × 15°, with the exception of the central four positions, which were exclusively reserved for the fixation point. Each quadrant of the matrix contained three items. All stimuli were black, presented against a mid-level gray background.

#### Design and procedure

Each trial commenced with a fixation display lasting between 900 and 1,100 ms, with the duration randomly drawn from a rectangular distribution. The fixation point, a combination of a bulls-eye and crosshair measuring 0.55° × 0.55°, was consistently used to minimize involuntary eye movements (Thaler et al., 2013). The search display remained on the screen until participants made a manual response or until 10 s elapsed without a response. Following this, a blank screen appeared for 500 ms before the onset of the next trial.

The main experiment consisted of 360 trials, divided into ten blocks. The first eight blocks functioned as a training session and featured an uneven spatial distribution of the target. In these blocks, the target appeared in a randomly selected *rich quadrant* in 50% of the trials. In the remaining trials, it was randomly presented in one of the other three quadrants, with each quadrant containing the target in 16.7% of the trials. The rich quadrant remained constant throughout the training and was counterbalanced across participants. Participants were not informed about the uneven distribution of the target. Half of the participants were instructed to use their right index finger to press key N for a 0° T and their right middle finger to press key M for a 180° rotated T. The other half used their left index finger to press key W for a 0° T and their left middle finger to press key Q for a 180° T. Given that response keys and rich quadrants had no significant impact on performance (*p*s > 0.055), except for a notable effect of rich quadrants on RTs in the *switch* group (*F*(3, 19) = 7.28, *p* = 0.002), data from these conditions were combined to enhance statistical power.

In the last two blocks, the testing session, the same uneven spatial distribution was maintained for all subjects in both groups. However, in the *switch* group, participants who had used their right hand were asked to switch to their left hand, pressing key W and Q for target identification, and vice versa for those who had used their left hand. In the *same* group, participants continued using their original response keys.

Participants received feedback about their average performance every two blocks. Prior to the main experiment, they received instructions and underwent a 9-point calibration of the eye-tracker, followed by 12 practice trials that maintained the same uneven spatial priority as in the training session of the main experiment. At the end of the experiment, participants were presented with a five-alternative forced-choice question. They were asked to indicate whether the target appeared with equal probability across all quadrants, or whether it appeared more frequently in the upper left, upper right, lower left, or lower right quadrants.

#### Statistical analysis

The overall accuracy was high in both groups (*same*: *M* = 0.99, *SD* = 0.01, *switch*: *M* = 0.99, *SD* = 0.01). Consequently, our primary analysis focused on RTs and eye-tracking data. Trials were excluded if participants’ RTs fell outside three standard deviations from their mean. Additional exclusion criteria included: trials with gaze deviation beyond the 3° × 3° fixation area before the appearance of the search array; trials where saccades failed to land on the target area, defined as instances where the distance between the fixations and the center of the target exceeded 1.5°. After applying these criteria, 6.27% of the data from the *same* group and 7.25% of the data from the *switch* group were excluded from the analysis. We also provided the Bayes factors (BFs) that quantify the relative likelihood of obtaining the observed data under the null hypothesis compared to the alternative hypothesis (Rouder et al., 2009). Evidence in favor of the null hypothesis is denoted as BF_01_, and in favor of the alternative hypothesis as BF_10_.

To analyze search behavior, we employed three dependent measures: *RTs*, *scan-path ratios*, and *fixation dwell times*. *RTs* acted as an overall measure of visual search efficiency. Further, we dissected search behavior into stages of attentional guidance and decision-making through scan-path ratios and fixation dwell times (Hout & Goldinger, 2015; Yu et al., 2022). *Scan-path ratios* were calculated by summing the amplitudes of all saccades (measured in degrees of visual angle) made before fixating on the target and dividing this sum by the shortest possible distance from the central fixation point to the target. A ratio of 1 indicates perfect attentional guidance (i.e., a pop-out effect), while ratios greater than 1 suggest less efficient guidance due to visits to non-target locations first. Additionally, we analyzed the landing quadrant of the first saccade as a complementary measure of attentional guidance. The results aligned with the scan-path ratio findings and are therefore presented in the Online Supplementary Material (Figs. S1 and S2). *Fixation dwell times* were assessed by measuring the total time spent fixating on the target, providing insight into the duration required to recognize the object as the target. Although the logic behind interpreting the timing of these events is intuitive, we also have empirical evidence supporting this distinction between metrics. Previous work showed that scan-path ratios are sensitive to how precisely subjects can use target features to guide attention, whereas dwell times selectively measure how difficult it is to decide that the fixated item is the target you are looking for (Hout & Goldinger, 2015; Yu et al., 2022).

### Results

#### Training

**Fig. 2:**
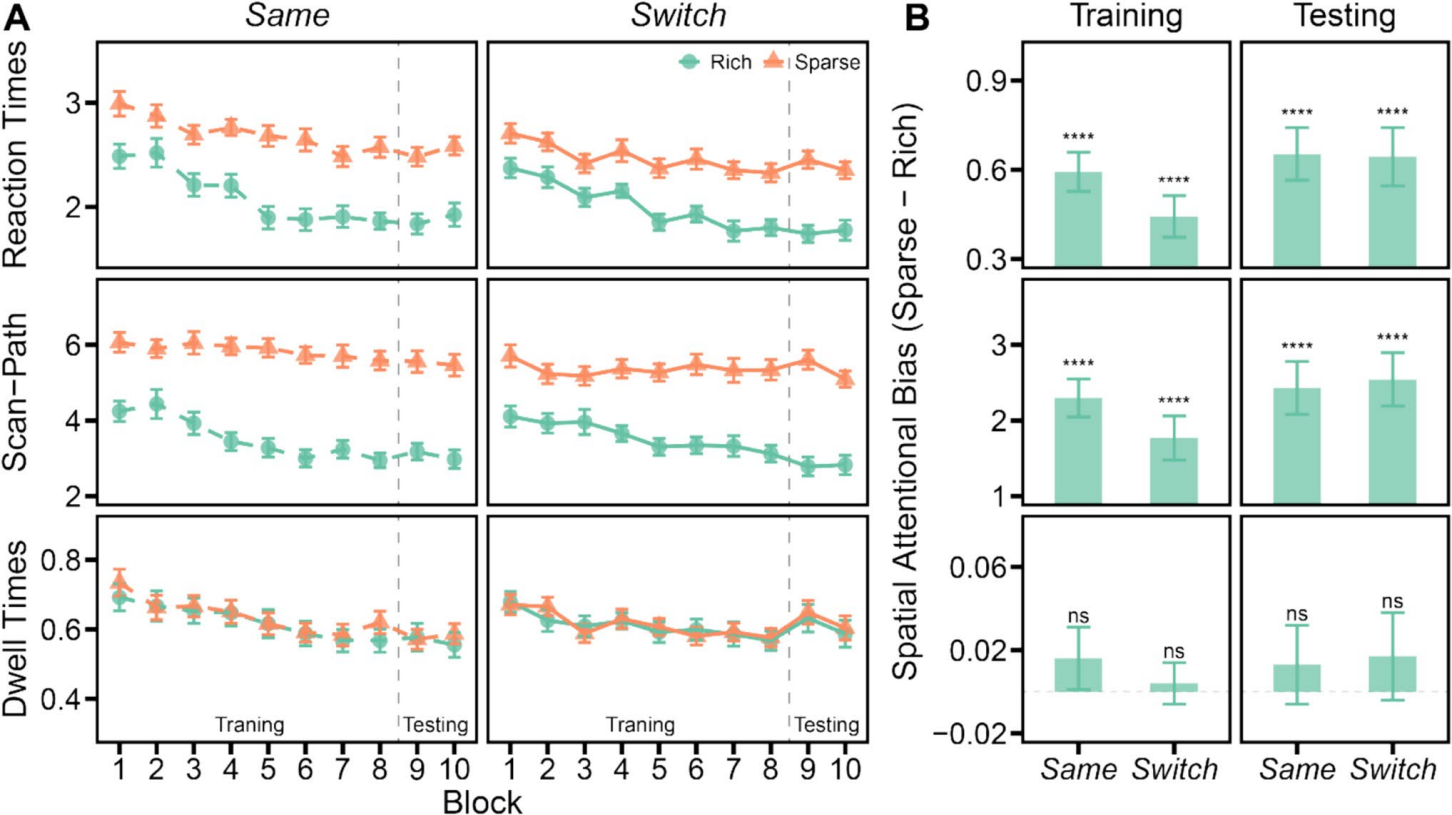
(**A**) This panel displays the results for three key dependent measures across ten experimental blocks: reaction times (RTs), scan-path ratios, and fixation dwell times. Data are categorized by quadrant type and participant group, with quadrant types identified by color (green for rich, orange for sparse) and participant groups distinguished by their position on the graph (left for the *same* group, right for the *switch* group). (**B**) This panel illustrates the spatial attentional bias, calculated by contrasting each dependent variable when the target was in sparse versus rich quadrants. In the training phase, significant positive biases in RTs and scan-path ratios across both groups reveal a preference for the rich quadrant. The absence of a significant bias in fixation dwell times indicates that the attentional bias did not enhance target identification speed. In the testing phase, the persistence of biases in RTs and scan-path ratios, consistent across groups, implies that adjustments to response features had no impact on the learned spatial priority. Error bars indicate the SEMs. **** *p* < 0.0001, ns *p* > 0.05

Figure 2A presents the results for three dependent measures: RTs, scan-path ratios, and fixation dwell times, across ten experimental blocks. These variables are differentiated by quadrant type (rich vs. sparse) and participant group (*same* vs. *switch*). To assess spatial attentional bias, we calculated the difference in each dependent variable when the target was located in the sparse quadrants versus in the rich quadrant. The biases observed during the training session, as shown in Fig. 2B, were analyzed using a one-sample *t*-test for each group.

Search RTs were significantly slower when the target was located in the sparse quadrants compared to the rich quadrant for both groups (*same*: *t*(23) = 8.95, *p* < 0.0001, *d* = −1.83, BF_10_ > 1,000; *switch*: *t*(23) = 6.31, *p* < 0.0001, *d* = 1.29, BF_10_ > 1,000). Additionally, significantly higher scan-path ratios were observed for targets in the sparse quadrants as opposed to the rich quadrant across both groups (*same*: *t*(23) = 9.10, *p* < 0.0001, *d* = 1.86, BF_10_ > 1,000; *switch*: *t*(23) = 6.06, *p* < 0.0001, *d* = 1.24, BF_10_ > 1,000), indicating a learned preference for the rich quadrant. However, the dwell times required to identify the target within the rich versus sparse quadrants did not significantly differ (*same*: *t*(23) = 1.05, *p* = 0.30, BF_01_ = 2.84; *switch*: *t*(23) = 0.36, *p* = 0.72, BF_01_ = 4.38). This finding suggests that while spatial attention was preferentially directed towards the rich quadrant, this did not facilitate faster target identification within that quadrant (Sha et al., 2018). Crucially, no significant differences in the spatial attentional bias were detected between the groups (*t*s < 1.56, *p*s > 0.12, BF_01_ > 1.30), indicating that the degree of bias towards the rich quadrant was consistent across groups.

#### Testing

If swapping response features was capable of resetting the learned priority, we would expect to see a rapid diminishment of the search advantage in the rich quadrant during the testing session once the *switch* group changed their response keys. Contrary to this expectation, the spatial attentional bias in RTs (*same*: *t*(23) = 7.42, *p* < 0.0001, *d* = 1.51, BF_10_ > 1,000; *switch*: *t*(23) = 6.57, *p* < 0.0001, *d* = 1.34, BF_10_ > 1,000) and scan-path ratios (*same*: *t*(23) = 6.96, *p* < 0.0001, *d* = 1.42, BF_10_ > 1,000; *switch*: *t*(23) = 7.22, *p* < 0.0001, *d* = 1.47, BF_10_ > 1,000) persisted for both groups throughout the testing session. Moreover, no significant differences were observed between the groups (*t*s < 0.22, *p*s > 0.82, BF_01_ > 3.41), including during block 9, the initial block in which the *switch* group utilized new response keys (*t*s < 0.81, *p*s > 0.42, BF_01_ > 2.66). These results suggest that changing response features did not negate the learned attentional bias.

#### Awareness

The final question served as a basic indicator of participants’ explicit awareness of the spatial distribution of targets. Thirteen out of 24 participants in the *same* group and 11 out of 24 participants in the *switch* group correctly identified the rich quadrant. Subsequently, the groups were divided based on their awareness reports, and the spatial attentional bias during the testing session was compared separately among participants who were aware and those who were not. No significant differences were observed between groups, regardless of the participants’ awareness of the spatial distributions (aware: *t*s < 1.73, *p*s > 0.1, BF_01_ > 0.93; unaware: *t*s < 1.21, *p*s > 0.24, BF_01_ > 1.58). This indicates that the observed effect was not influenced by the participants’ explicit knowledge of the statistical regularity.

## Experiment 2

The findings from Experiment 1 showed that modifying response configuration did not undermine the learned attentional bias. In Experiment 2, we used a novel introduction of a rich-second quadrant in the test phase. We hypothesized that altering response requirement might facilitate the formation of a new association between the now-prioritized rich quadrant and its corresponding response features. According to this hypothesis, we expected the *switch* group to demonstrate an enhanced ability to shift their spatial attention effectively towards this newly enriched quadrant. Therefore, in Experiment 2, we aimed to observe the benefits of changing response features, in contrast to our previous investigation in Experiment 1, where we focused on potential interference resulting from such response changes.

### Material and methods

#### Participants

Forty-eight undergraduates from Vanderbilt University participated in this study for course credit (33 self-reported females, 15 males 15; age range: 18–24 years). They were randomly assigned to either the *switch* or the *same* groups. All participants provided written consent after being informed and reported having either normal or corrected-to-normal vision.

#### Design and procedure

The methodology for this experiment followed that of Experiment 1, with a key modification: the introduction of a new *rich-second quadrant* for the testing session (Fig. 1B). This quadrant, positioned diagonally opposite the *rich-first quadrant* from the training session, became the primary target location during the testing phase, with the target appearing there in 50% of the trials. In contrast, the original rich-first quadrant and the remaining sparse quadrants each contained the target in 16.7% of trials. Similar to Experiment 1, participants in the *switch* group were instructed to change their response hands and keys for the testing session, while the *same* group continued with their initial response configuration. At the experiment’s end, participants were asked to answer two five-alternative forced-choice questions to identify the rich quadrant for both the training and testing phases.

#### Statistical analysis

The overall accuracy was high in both groups (*same*: *M* = 0.99, *SD* = 0.01, *switch*: *M* = 0.99, *SD* = 0.01). Consequently, the analyses primarily concentrated on RTs and eye-tracking data. Following the same criteria as in Experiment 1, we excluded 8.61% of the data from the *same* group and 8.58% of the data from the *switch* group.

### Results

#### Training

**Fig. 3:**
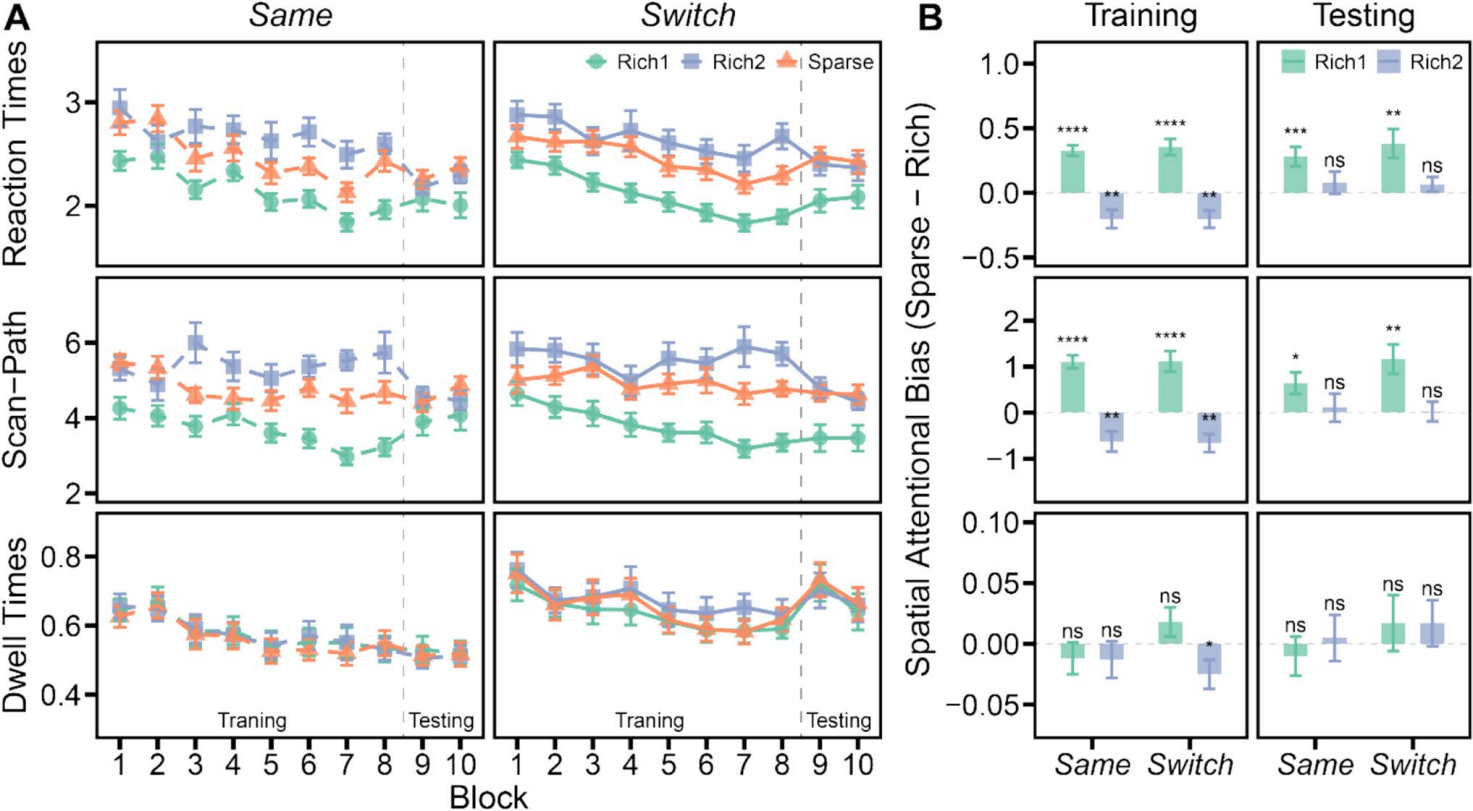
(**A**) This panel displays the results for three key dependent measures across ten experimental blocks: reaction times (RTs), scan-path ratios, and fixation dwell times. Data are categorized by quadrant type and participant group, with quadrant types identified by color (green for rich-first, purple for rich-second, orange for sparse) and participant groups distinguished by their position on the graph (left for the *same* group, right for the *switch* group). (**B**) This panel illustrates the spatial attentional biases, measured by contrasting each dependent variable for targets appearing in sparse versus the two rich quadrants. During the training phase, both groups exhibited significant positive biases in RTs and scan-path ratios towards the rich-first quadrant, which had a 50% likelihood of containing the target. In contrast, negative biases for the rich-second quadrant imply that participants’ search behaviors conformed to routine scanning patterns, initially focusing on the rich-first quadrant, subsequently scanning the adjacent sparse quadrants, and ultimately moving to the diagonally opposite rich-second quadrant. During the testing session, despite the relocation of the 50% likelihood target-containing quadrant from the rich-first to the rich-second quadrant, participants in both groups maintained their preference for the rich-first quadrant, indicating a slow adjustment of bias towards the currently rich quadrant. The lack of significant differences between the groups indicates that switching response features did not effectively facilitate the relearning of spatial priorities. Error bars indicate the SEMs. **** *p* < 0.0001, *** *p* < 0.001, ** *p* < 0.01, * *p* < 0.05, ns *p* > 0.05

Figure 3A illustrates the outcomes for each dependent variable across ten experimental blocks, differentiated by quadrant type and participant group. Consistent with Experiment 1, we calculated the difference in performance when the target was located in the sparse quadrants versus the two rich quadrants (Fig. 3B) and conducted a one-sample *t*-test to assess statistical significance.

When the target was located in the rich-first quadrant, which had a 50% likelihood of containing targets, both groups demonstrated faster search RTs (*same*: *t*(23) = 8.07, *p* < 0.0001, *d* = 1.65, BF_10_ > 1,000; *switch*: *t*(23) = 5.64, *p* < 0.0001, *d* = 1.15, BF_10_ > 1,000) and reduced scan-path ratios (*same*: *t*(23) = 7.86, *p* < 0.0001, *d* = 1.60, BF_10_ > 1,000; *switch*: *t*(23) = 4.98, *p* < 0.0001, *d* = 1.02, BF_10_ > 1,000). However, no significant differences were observed in fixation dwell times between targets in the rich-first versus sparse quadrants (*same*: *t*(23) = −0.94, *p* = 0.36, BF_01_ = 3.12; *switch*: *t*(23) = 1.48, *p* = 0.15, BF_01_ = 1.79). Importantly, no significant differences were detected in spatial attentional bias between the groups (*t*s < 1.69, *p*s > 0.10, BF_01_ > 1.10), underscoring the effectiveness of both groups in learning which quadrant was more likely to contain the target.

When the target appeared in the rich-second quadrant, positioned diagonally opposite the rich-first quadrant with only a 16.7% chance of containing targets, both groups exhibited longer RTs (*same*: *t*(23) = −2.87, *p* = 0.009, *d* = −0.59, BF_10_ = 5.46; *switch*: *t*(23) = −3.12, *p* = 0.005, *d* = −0.64, BF_10_ = 8.90) and larger scan-path ratios (*same*: *t*(23) = −2.86, *p* = 0.009, *d* = −0.58, BF_10_ = 5.38; *switch*: *t*(23) = −3.30, *p* = 0.003, *d* = −0.67, BF_10_ = 12.93). These results align with the routinized scanning procedures (Seitz et al., 2023), indicating that participants initially focused on the rich-first quadrant, then proceeded to check the adjacent sparse quadrants, and finally moved to the diagonally opposite rich-second quadrant. Additionally, the *switch* group showed slightly longer fixation dwell times (25 ms) when the target was in the rich-second quadrant compared to the sparse quadrants (*t*(23) = −2.13, *p* = 0.044, *d* = −0.43, BF_10_ = 1.44); this pattern was not evident in the *same* group (*t*(23) = −0.85, *p* = 0.4, BF_01_ = 3.36). However, the overall non-significant differences in fixation dwell times suggest that the attentional bias did not facilitate target identification. Furthermore, no significant differences between groups were observed (*t*s < 0.63, *p*s > 0.53, BF_01_ > 2.96).

#### Testing

In the testing session, the quadrant with a 50% likelihood of containing the target was changed from the initial rich-first quadrant to the diagonally opposite, rich-second quadrant. We hypothesized that if changing response requirement facilitated the formation of a new association between the now-prioritized rich-second quadrant and its response characteristics, then the *switch* group would be more effective in reallocating spatial attention towards this quadrant compared to the *same* group. Consequently, we expected the interference effect associated with the previously prioritized rich-first quadrant to be less pronounced in the *switch* group. Moreover, we anticipated a more robust attentional bias towards the rich-second quadrant within the *switch* group.

Participants in both groups demonstrated a persistent attentional bias towards the rich-first quadrant, as evidenced by shorter search RTs (*same*: *t*(23) = 3.81, *p* = 0.001, *d* = 0.78, BF_10_ = 38.30; *switch*: *t*(23) = 3.42, *p* = 0.002, *d* = 0.70, BF_10_ = 16.85) and smaller scan-path ratios (*same*: *t*(23) = 2.78, *p* = 0.011, *d* = 0.57, BF_10_ = 4.55; *switch*: *t*(23) = 3.65, *p* = 0.001, *d* = 0.75, BF_10_ = 27.22). Although RTs in the rich-second quadrant improved in the testing session compared to the training session (*same*: *t*(23) = −3.26, *p* = 0.003, *d* = −0.66, BF_10_ = 11.87; *switch*: *t*(23) = −4.43, *p* = 0.0002, *d* = −0.91, BF_10_ = 151.78), they were not significantly faster than those in the sparse quadrants (*same*: *t*(23) = 0.91, *p* = 0.37, BF_01_ = 3.21; *switch*: *t*(23) = 1.14, *p* = 0.26, BF_01_ = 2.60). Similarly, scan-path ratios in the testing session were reduced (*same*: *t*(23) = −2.26, *p* = 0.034, *d* = −0.46, BF_10_ = 1.79; *switch*: *t*(23) = −2.73, *p* = 0.012, *d* = −0.56, BF_10_ = 4.14), yet they did not significantly differ from those in the sparse quadrants (*same*: *t*(23) = 0.38, *p* = 0.71, BF_01_ = 4.37; *switch*: *t*(23) = 0.13, *p* = 0.9, BF_01_ = 4.62). Importantly, there were no significant differences between the groups (*t*s < 1.33, *p*s > 0.19, BF_01_ > 1.70). These findings imply that the attentional bias acquired during the training phase interfered with the acquisition of a new bias towards the currently rich quadrant, and that merely switching response features was insufficient to prompt a recalibration of the attentional bias.

#### Awareness

Three out of 24 participants in the *same* group and one out of 24 participants in the *switch* group successfully identified the rich quadrant in both the training and testing sessions. Furthermore, ten participants from each group were able to identify the rich quadrant in only one session. These participants were collectively referred to as the *aware participants*. The analysis of spatial attentional bias during the testing session revealed no significant differences between groups, irrespective of participants’ awareness of spatial distributions (aware: *t*s < 2.01, *p*s > 0.06, BF_01_ > 0.65; unaware: *t*s < 0.79, *p*s > 0.44, BF_01_ > 2.13). These findings suggest that explicit knowledge of the statistical regularities did not influence the overall pattern of results.

## General discussion

When participants are exposed to a task, each repetition is stored as a separate episode in memory, encompassing stimuli, responses, and outcomes. With repeated exposure to the same task, participants amass a vast repository of these episodes. This leads to more efficient retrieval of these episodes with each repetition, especially under consistent task conditions. Such efficiency translates into faster responses that we describe as a state of automaticity (Logan, 1988, 2002; Logan & Bundesen, 2004). In the context of the probability cueing paradigm, where a target appears more frequently at a specific location, every successful detection of the target at that location results in the storage of another episodic memory trace for that particular task. As these episodes accumulate, they race for retrieval and the minimum finishing time to retrieve the behavioral response decreases with more runners in the race (i.e., more trials of experience). This is how the instance theory of automaticity accounts for our cognitive systems becoming more adept at quickly accessing the relevant information needed to respond to the target in that location again. This occurs because the frequent appearance of the target at the same location increases the predictability of the task, allowing for automatic long-term memory retrieval of the correct behavioral response. With sufficient practice, the search task can be performed rapidly and with minimal working memory (Gao & Theeuwes, 2020b; Won & Jiang, 2015) or executive control demands (Gao & Theeuwes, 2020a), relying instead on the rapid retrieval of episodes from long-term memory. The increased speed at which participants detect a target in a frequently encountered location is thus a manifestation of the statistical learning effect.

The current study explored the role of episodic retrieval in visual statistical learning, with a particular focus on how changes in response features affect the retrieval of memory episodes and, consequently, the learned attentional bias. Previous research suggests that altering target-defining features can reset established spatial priorities (Addleman et al., 2019; Zhang & Carlisle, 2023), presumably because such changes prevent the retrieval of relevant memory episodes. Building on this foundation, we examined whether changes in motor responses could negate the statistical learning effect. Contrary to our initial hypothesis, results from two experiments indicated that modifying response features did not alter the spatial attentional bias. Specifically, in Experiment 1, even after changing response keys, the *switch* group continued to preferentially direct their attention to the quadrant where targets had been more frequently observed. Similarly, in Experiment 2, when a new rich quadrant was introduced during the testing session, the *switch* group did not develop a stronger bias towards this newly prioritized quadrant. These findings indicate that simply changing response features is insufficient to either disrupt an existing attentional bias or foster a new one. We discuss three possible explanations for these null results in the sections that follow.

One possibility is that memory episodes selectively encode properties crucial for target selection, such as the target’s defining features and location, while omitting response features and elements irrelevant to the task (Maljkovic & Nakayama, 1994, 1996). Furthermore, the episodic-retrieval process may be more influenced by the general context of the memory episode rather than specific details. For example, Lamy et al. (2011) found that response repetition quickened the search process when both the target and distractor colors were the same as in previous trials. In contrast, the search slowed when the colors of the target and distractors were swapped, yet there was no effect on performance when the color of either the target or the distractors was changed to a new color. This suggests that the retrieval process is shaped by the overall configuration of the memory episode, rather than by isolated changes within it.

A second possibility is that episodic retrieval may primarily influence stages of the search process that occur after the competition for attention has been resolved, particularly target identification and response execution (Lamy et al., 2010; Yashar & Lamy, 2011; Zehetleitner et al., 2012). The recent study by Theeuwes et al. (2024) revealed that participants could learn that a specific response is more likely needed when the singleton target appears at a certain location. However, the singleton target is equally probable to appear at any location; the sole contingency is that if the target appears at one particular location, one response becomes more probable than the other. This suggests that the effect observed is a bias in a later stage, either in the identification of reported features (i.e., distinguishing a horizontal or vertical line) or in the selection of a motor response (i.e., pressing an up or left response key) (see also Allenmark et al., 2024). Similarly, Hilchey et al. (2018) proposed that when the target location repeats, the response recently associated with that location is quickly retrieved and reactivated, after attention has been directed to that location. Nevertheless, in our experiments, probability cueing resulted only in an attentional bias towards the rich quadrant and did not facilitate faster target identification compared with the sparse quadrants.

Third, perhaps we observed no effect when we changed the response buttons because participants viewed their primary motor response as making eye movements to the target. If participants are storing stimulus–response pairings of the object and the eye movement they made to it, then we would expect exactly the pattern of results we just found, where the button press was not part of the stored instance of performance. This account would still be consistent with the instance theory of automaticity and the proposal that we might have seen effects if we had made the task an anti-saccade task in the switch conditions of the present study. We cannot rule out this proposal with our existing findings, although previous studies suggest that eye-movement behavior does not change when target locations are statistically predictable (McDonnell et al., 2015). This idea is directly empirically testable in future work.

Finally, visual statistical learning might not depend on episodic retrieval but instead operates via the continuous adjustment of weights within a spatial priority map. According to this framework, locations that previously contained targets are assigned higher priority weights, while those more likely to harbor distractors see a reduction in priority. Consequently, the process of attentional selection is determined by this evolving priority map, which synthesizes various signals including top-down search goals, bottom-up salience of stimuli, and the history of past selections. Thus, the statistical learning of target locations can be understood as reflecting changes in the weights assigned on spatial priority maps (Theeuwes et al., 2022).

Statistical learning is a complex process, likely incorporating multiple distinct cognitive mechanisms. In our study, we aimed to explore the contribution of episodic retrieval to the probability cueing effect. Our findings allow us to rule out the possibility that modifying response features resets our acquisition of attentional bias, thereby adding valuable insights to the burgeoning body of research on the role of episodic retrieval in statistical learning (Lamy et al., 2024).

## Supplementary Information

Below is the link to the electronic supplementary material.

Supplementary file1 (DOCX 471 KB)

## Data Availability

The processed subject level data are provided via the Open Science Framework at: https://osf.io/ygwxu/.

## Code availability

The analysis code is provided via the Open Science Framework at: https://osf.io/ygwxu/.

## Declarations

### Open practices statement

The processed subject level data and analysis code are provided via the Open Science Framework at: https://osf.io/ygwxu/.
