# Supplementary material for: Investigating memory episodes in location probability learning: Can altering response features reset spatial bias?
Source: Atten Percept Psychophys. 2025 Jun 11;87(5):1518–29. doi: 10.3758/s13414-025-03106-6 (PMC12204878; doi:10.3758/s13414-025-03106-6)
Supplement: Supplementary file 1 — Supplementary file1 (DOCX 471 KB) [file 13414_2025_3106_MOESM1_ESM.docx]

**Supplemental Materials**

In Experiment 1, we analyzed the landing quadrant of the first saccade as an additional measure of attentional guidance, alongside scan-path ratios. To assess spatial attentional bias, we calculated the difference in the probability of the first saccade landing in the rich quadrant compared to the sparse quadrants. The biases observed during the training session, presented in Figure S1, were analyzed using a one-sample *t*-test for each group. The results showed that the probability of the first saccade landing in the rich quadrant was significantly higher than the probability of landing in the sparse quadrants for both groups (*same*: *t*(23) = 9.20, *p* < .0001, *d* = 1.88, BF_10_ > 1000; *switch*: *t*(23) = 5.66, *p* < .0001, *d* = 1.16, BF_10_ > 1000). These findings indicate a learned preference for the rich quadrant. Additionally, participants in the *same* group demonstrated a stronger spatial attentional bias toward the rich quadrant compared to participants in the *switch* group, *t*(24) = 2.00, *p* = .05, *d* = 0.58, BF_10_ = 1.42.

During the testing session, the spatial attentional bias in the first saccade persisted for both groups (*same*: *t*(23) = 9.87, *p* < .0001, *d* = 2.01, BF_10_ > 1000; *switch*: *t*(23) = 7.86, *p* < .0001, *d* = 1.60, BF_10_ > 1000). These findings are consistent with the scan-path ratio results, suggesting that altering response features did not disrupt the learned attentional bias.


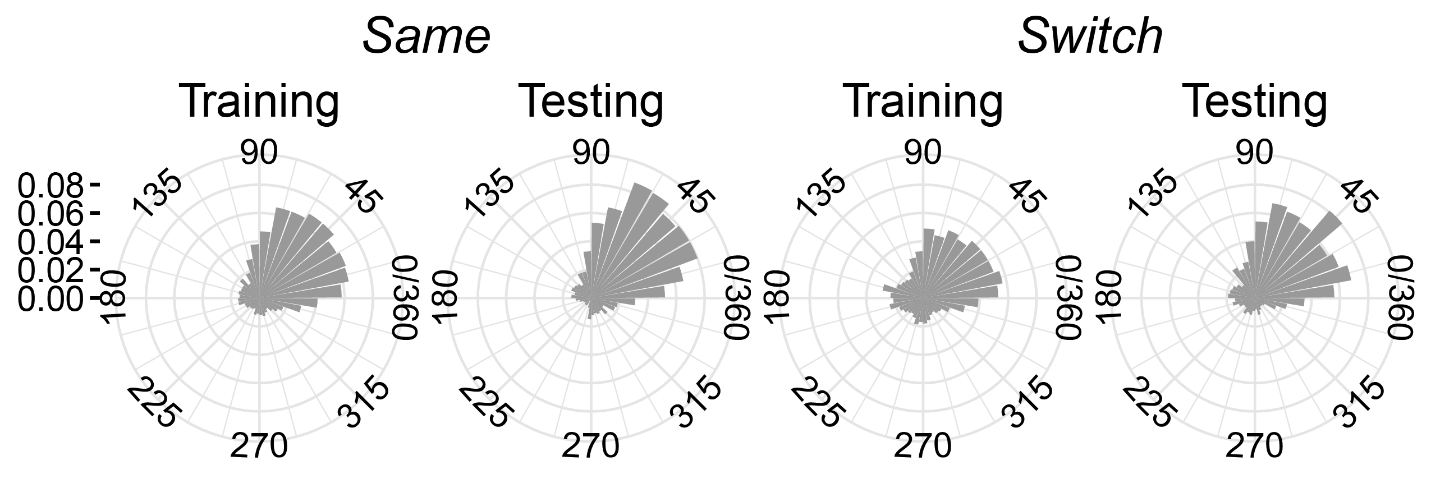


*Figure S1*. The polar histogram depicts the visual angle of the first saccade in Experiment 1, separated by experimental session and participant group. For visualization purposes, we collapsed across the rich quadrants, assigning Quadrant 1 (0° to 90°) as the rich quadrant. Participants in both groups demonstrated a spatial attentional bias toward the rich quadrant, and altering response methods did not eliminate this learned bias.

Next, we analyzed the landing quadrant of the first saccade as an additional measure of attentional guidance in Experiment 2. Both groups made significantly more first saccades toward the rich-first quadrant (*same*: *t*(23) = 7.53, *p* < .0001, *d* = 1.54, BF_10_ > 1000; *switch*: *t*(23) = 7.10, *p* < .0001, *d* = 1.45, BF_10_ > 1000), highlighting that both groups learned which quadrant was more likely to contain the target.

During the testing session, the quadrant with a 50% likelihood of containing the target was shifted from the original rich-first quadrant to the diagonally opposite rich-second quadrant. Despite this change, participants in both groups exhibited a persistent attentional bias toward the rich-first quadrant, (*same*: *t*(23) = 2.66, *p* = .01, *d* = 0.54, BF_10_ = 3.68; *switch*: *t*(23) = 3.41, *p* = .002, *d* = 0.70, BF_10_ = 16.34). Participants in the *same* group also showed a stronger attentional bias toward the rich-second quadrant, *t*(22) = 3.26, *p* = .004, *d* = 0.68, BF_10_ = 11.78, suggesting that they quickly adapted to the current high-probability target location. In contrast, participants in the *switch* group exhibited only a numerical increase in first saccades to the rich-second quadrant (27%) compared to the sparse quadrants (20%), which were not statistically significant (*t*(23) = 1.51, *p* = .15, BF_01_ = 1.73). Notably, there was no significant difference between the groups (BF_01_ > 3.00). These findings align with the scan-path ratio results, indicating that merely switching response features was insufficient to recalibrate the attentional bias.


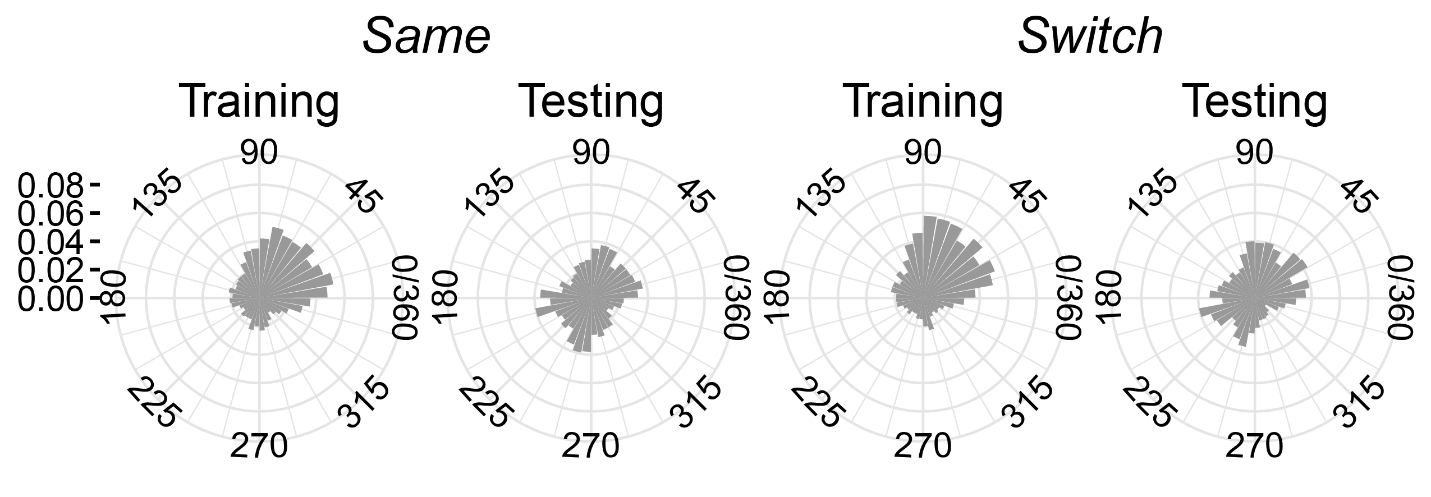


*Figure S2*. The polar histogram illustrates the visual angle of the first saccade in Experiment 2, categorized by experimental session and participant group. For visualization purposes, we collapsed across the rich quadrants, designating Quadrant 1 (0° to 90°) as the rich quadrant. During the testing session, the quadrant with a 50% likelihood of containing the target shifted from Quadrant 1 to the diagonally opposite Quadrant 3 (180° to 270°). Participants in both groups exhibited a spatial attentional bias toward the rich-second quadrant during the testing phase. However, the absence of a significant between-group difference suggests that merely switching response features did not enhance the recalibration of the attentional bias.
